# The Secret of Good Manners

**Published:** 1881-06

**Authors:** 


### THE SECRET OF GOOD MANNERS.

The secret of good manners is to forget one's own self altogether. The people of really fine breeding are the ones who never think of themselves, but only of the pleasure they can give to others. No adornment of beauty, or learning, or accomplishments, go so far in its power to attract as the one gift of sympathy.

In all French history no woman had a stronger fascination for whoever came within her reach than Madame Recamier. She was called beautiful; but her portraits prove that her beauty was not to be compared with that of many less charming women. And when every attraction of person had long since passed away and she was an old, old woman, her sway over the hearts of others was as powerful as ever. What was her secret ?

It was this one thing solely—her genuine and unaffected interest in the good and ill fortunes of her friends. Authors came to her and read her their books; painters came to her with their pictures; statesmen with their projects. She herself wrote no books, painted no pictures, had no projects. She was sweet, simply and unconsciously, as a rose is sweet. She really cared for the happiness and success of others, and they felt the genuineness of her sympathy. It surrounded her with an immortal charm.

Let any girl try Madame Recamier's experiment. Let her go into society thinking nothing of the admiration she may win; but everything of the happiness she may confer. It matters little whether her face is beautiful, or her toilette costly. Before the end of three months she will be a happy girl herself, for the world likes sunshine and sympathy, and turns to them as the flowers bask in the sun of June.

